# Insights from genetically stratified analyses comparing subtypes of alcohol misuse

**DOI:** 10.1177/00368504241260375

**Published:** 2024-06-11

**Authors:** Jeanne E Savage, Anaïs B Thijssen

**Affiliations:** 1 Department of Complex Trait Genetics, Center for Neurogenomics and Cognitive Research, 1190 Vrije Universiteit Amsterdam, Amsterdam Neuroscience, Amsterdam, The Netherlands; 2 Department of Psychiatry, Genetic Epidemiology, Amsterdam UMC location University of Amsterdam, Amsterdam, The Netherlands; 3 Amsterdam Neuroscience, Amsterdam Public Health, Amsterdam, The Netherlands

**Keywords:** Alcohol misuse, latent class, heterogeneity, GWAS, typology

## Abstract

In a recent publication, we applied a novel model to address phenotypic heterogeneity in genetic research on alcohol misuse by stratifying individuals based on their patterns of alcohol use behaviours and comorbid psychopathology. In this Commentary, we provide further descriptions of the subtypes of alcohol misuse that emerged from the empirical mixture modelling approach and present new results comparing these groups on sociodemographic characteristics and additional alcohol use outcomes. We take a broad perspective to discuss how these results fit with existing typologies of alcohol misuse and how the results inform future genetic research. Our findings add further evidence that conceptualisations of a binary distinction between ‘internalising’ (relief-seeking) versus ‘externalising’ (reward-seeking) subtypes does not fully capture the complexity of alcohol misuse. However, accounting for individual differences in these dimensions is a promising means to reduce heterogeneity and thereby improve power for gene discovery and, eventually, personalised medicine applications. We argue that more detailed, person-specific assessment of alcohol misuse measures, particularly with attention to longitudinal trajectories, is needed to further advance this important line of research.

## Overview

Our recent study^1^ represents one of the few existing attempts to incorporate the well-known phenotypic heterogeneity of alcohol misuse^2^ into large-scale genetic association (GWAS) research. Using a mixture modelling approach, we empirically identified four subgroups of adults based on their distinct patterns of alcohol use behaviours and comorbid psychopathology. These included a group with low levels of all psychopathology and alcohol misuse (‘low risk’), a group with anxiety and depression symptoms but little current alcohol use (‘internalising’) and two groups with similarly high levels of alcohol use but of which only one had high rates of alcohol use disorder (AUD) diagnoses and other psychopathology (‘heavy use, low problems’ vs. ‘broad risk’ groups). Using GWAS and bioinformatics tools, we identified numerous genetic differences between the groups. This multidimensional approach generated advances in gene discovery for alcohol misuse over previous studies using simpler measures such as AUD diagnoses or overall alcohol consumption levels. In this Commentary, we will expand upon the relevance of the modelling approach for uncovering the etiology of alcohol misuse and provide further results to support an understanding of the similarities and differences between groups.

## Methodology

As previously described,^1^ data were drawn from *N *= 410,414 individuals from the UK Biobank, a population-based cohort of adults in the UK with a broad array of assessments including surveys, biological samples and linked medical records. Each individual was assigned to one of four latent classes (low risk, *n *= 105,142; internalising, *n *= 125,318, heavy use, *n *= 94,731 and broad risk, *n *= 85,770) based on their patterns of self-reported symptoms/behaviours and recorded diagnoses related to internalising, externalising and alcohol use disorders. Mixture modelling was carried out in Mplus^3^ to identify the best fitting model and match individuals to a latent class based on their highest posterior probability of class membership. In the current study, we make use of the previously derived latent classes to further characterise the similarities and differences between these groups. We compared the classes on 10 selected sociodemographic characteristics and alcohol-related diagnoses that were not included in the latent class definitions. Exploratory comparisons of the means/proportions between latent classes were conducted using ANOVA tests for continuous variables and χ^2^ tests for dichotomous variables, with a two-tailed Bonferroni-corrected significance threshold (*p *< .05/10 = .005). Furthermore, we repeated the previous mixture modelling approach^1^ (using the same methods and input variables) within two subsets of younger (age ≤ 55 at intake; *n *= 237107) and older (age > 55, n *= *173854) participants with the UK Biobank cohort in order to qualitatively compare the latent class patterns for indications of developmental trends. Results from these additional analyses are presented in context of the discussion of the original results, below.

## Internalising/externalising typology of alcohol misuse

Alcohol misuse frequently co-occurs with an array of different psychiatric disorders.^4^ This study was built upon a substantial body of theory and previous research pointing to an etiological distinction between ‘internalising’ drinkers, whose motivation stems from seeking relief from negative emotions, and ‘externalising’ drinkers, whose reward-seeking patterns of drinking are driven by behavioural undercontrol.^5–9^ Within this typology, internalising alcohol problems are thought to be more environmentally driven and externalising problems more heritable, making genetic research especially relevant. There is also evidence that *environmental* influences are responsible for the overlap between AUD and internalising spectrum disorders, while *genetic* influences account for the overlap between AUD and externalising spectrum disorders.^10^ We aimed to leverage this knowledge to improve power for gene discovery within these distinct subtypes.

In this new study,^1^ we did find evidence of an internalising class, but not one linked to alcohol problems. Rather, the internalising class had the highest proportion of lifelong abstainers and former drinkers and the lowest levels of current alcohol consumption. This would seem to suggest that a predisposition to internalising psychopathology (in the absence of other psychiatric disorders) may be protective against alcohol problems. Additionally, a specific externalising class did not emerge – although we should note that the externalising measures available in the UK Biobank sample were limited. Instead, we saw that alcohol problems were most prominent in the ‘broad’ class whose members exhibited both internalising and externalising psychopathology. The ‘heavy’ class additionally had high levels of alcohol use but remained relatively unburdened by other mental health problems. Although this class structure was not in line with the expected internalising/externalising typology, it is consistent with some studies that have found the groups to be more muddled or found that the *co-occurrence* of internalising and externalising symptoms is associated with alcohol misuse rather than wholly separate subtypes.^11,12^

In conjunction with the broader literature, our findings suggest that the internalising/externalising typology, while indexing important dimensions of individual variation, is not sufficiently nuanced to reflect the underlying etiology of alcohol misuse. In particular, this typology fails to capture longitudinal changes and (neuro)adaptations relevant to alcohol misuse. It is plausible that the latent class structure found in our study reflects the older age of the UK Biobank sample. An externalising subtype may have been evident had we looked earlier in life, before these individuals may have developed comorbid internalising psychopathology as a consequence of chronic heavy alcohol use. This would be consistent with the implications of the predominant model of the neurobiology of addiction,^13^ which suggests that withdrawal-driven relief-seeking (internalising) processes become salient after chronic alcohol consumption, even if the initial motivations were reward-seeking (externalising). Indeed, the internalising/externalising distinction is more clearcut in some younger samples,^7^ indicating that the inconsistency in support for this typology may be due to developmental changes in motivations for drinking across the lifespan.^14^ It may be the case that the observed ‘protective’ effect of internalising psychopathology is also an artefact of the sample age. If an internalising pathway to alcohol misuse is indeed more environmentally influenced,^10^ individuals in the internalising class might be more responsive to environmental moderators of alcohol use behaviours. In particular, this group could exhibit low current levels of alcohol use due to a sensitivity to negative alcohol-related consequences experienced earlier in life (perhaps more easily experiencing guilt or social pressures), spurring them to quit or reduce drinking. Such patterns cannot be accounted for with data that records only a snapshot of behaviour at one point in the lifespan.

**Figure 1.**
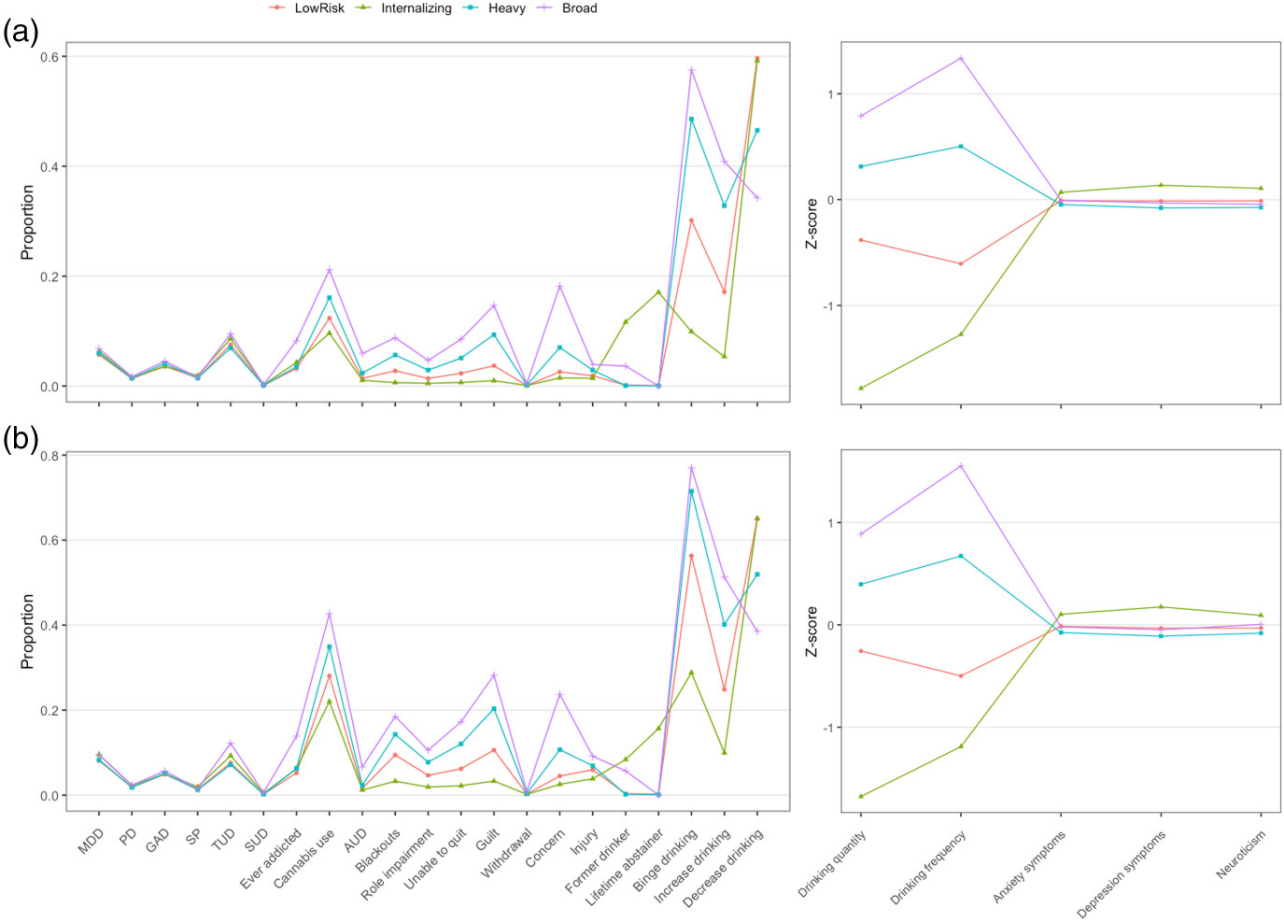
Mixture model results in (a) younger (age ≤ 55; *n *= 237,107) and (b) older (age > 55; *n = *173,854) individuals from the UK Biobank. Note: Probabilities and standardised mean differences are presented in separate panels. MDD: major depressive disorder; PD: panic disorder; GAD: generalised anxiety disorder; SP: specific phobia; TUD: tobacco use disorder; SUD: substance use disorder; AUD: alcohol use disorder.

To examine these possible alternative explanations, we carried out two exploratory analyses expanding on our previously published results. First, to investigate whether age differences may have biased the observed latent class structure, we stratified the UK Biobank data by age (older/younger than 55) and repeated the mixture modelling analysis. We found that the pattern of results was very consistent across both younger and older individuals (Figure 1). Second, we investigated whether the internalising group exhibited more characteristics of ‘responsiveness’ to negative consequences of their alcohol use by carrying out exploratory comparisons of the latent classes on specific alcohol-related diagnostic codes from lifetime medical records. ANOVA and chi-square tests (Table 1) showed that the internalising class had the lowest levels of recorded alcohol problems such as abuse or alcoholic liver disease, although they had the highest levels of a recorded ‘personal history of alcoholism’. This may indicate that high-internalising individuals are quicker to perceive their drinking as problematic and reduce alcohol use before physiological consequences manifest.

**Table 1.** Comparison of selected medical, environmental and demographic characteristics between classes.

Measure	Field code^a^	Mean (SD)/% endorsed				*F*/*χ^2^*	*P*-value
		Class 1 (low risk) *n *= 105,142	Class 2 (internalising) *n *= 125,318	Class 3 (heavy) *n *= 94,731	Class 4 (broad risk) *n *= 85,770		
Alcohol abuse or hazardous use	42040, 41270	0.25	0.14	0.37	0.93	5449	<1e-320
Alcoholic liver disease	42040, 41270	0.05	0.04	0.06	0.28	2086	<1e-320
Personal history of alcoholism	42040, 41270	3.21	3.84	2.98	3.64	1312	3.03E-284
Townsend Deprivation Index	22189	−1.44 (3.01)	−0.64 (3.36)	−1.77 (2.83)	−1.58 (2.94)	1288	7.88E-282
Post-secondary educational degree	6138	10.88	12.01	11.60	11.04	4504	<1e-320
Frequency of social visits (days/month)	1031	8.65 (8.58)	8.98 (9.23)	8.44 (8.35)	8.49 (8.73)	67.53	2.08E-16
Able to confide in someone	2110	13.81	14.67	13.12	11.70	1373	1.39E-297
Lifetime trauma exposure (any)	20487–20531	14.92	15.78	15.92	15.42	119	1.20E-25
Childhood abuse	20488, 20490	6.00	6.75	6.09	5.74	132	1.41E-28
Count of lifetime traumatic events	20487–20531	1.49 (1.92)	1.76 (2.26)	1.41 (1.76)	1.55 (1.87)	15.51	8.23E-05

a Field code from which the measures were derived, as listed in the UK Biobank showcase (https://biobank.ctsu.ox.ac.uk/showcase/). Two-tailed Bonferroni-corrected significance threshold = .005.

Our exploratory analyses, however, are limited by the age range (middle to older adulthood) and level of assessment detail available in UK Biobank. Formal comparisons using designs such as latent transition analysis in longitudinal samples are needed to explore how the internalising/externalising typology may manifest across salient developmental milestones starting in adolescence or younger adulthood. To fully answer these questions, a greater focus on trajectories of health and behaviour across the lifespan is essential, especially in genetic research of complex phenotypes.

## Genetic comparisons of subgroups

The characteristic that separates the study by Thijssen et al.^1^ from previous typologies of alcohol misuse is the comparison of groups on their underlying genetic differences. Using GWAS, we identified 96 regions of the genome at which allele frequencies differed significantly between latent classes, including many which were not found in previous studies of simpler, unidimensional measures of alcohol misuse. This study therefore highlighted the value of addressing phenotypic heterogeneity in genetic research. The strongest genetic signal distinguished the two low alcohol use classes (low risk; internalising) from the two high alcohol use classes (heavy; broad), most strongly broad from internalising. These genetic influences, however, did not map directly onto risk pathways earlier in life. Using a college student sample in which we previously observed distinct internalising and externalising subtypes of drinkers,^7^ we found that genetic variants associated with the broad latent class corresponded to the internalising subtype in young adulthood, but neither the broad nor heavy class showed a genetic overlap with the young adult externalising subtype. This finding may simply reflect the lack of externalising measures in the UK Biobank, but may again highlight the need to consider important etiological changes across the lifespan.

An additional finding of interest was the lack of any robust genetic effects distinguishing the heavy from the broad class. These are perhaps the most relevant classes to compare, as they differ primarily on clinically significant symptoms/disorders while both having high levels of alcohol consumption. All pairwise class comparisons had similar statistical power (80,000–110,000 individuals in each of the four groups), yet only for the comparison of the heavy and broad classes were no genome-wide significant loci found. This suggests that environmental exposures, in the context of a similar genetic predisposition to alcohol misuse, may determine the manifestation of clinically relevant psychopathology. Exploratory comparisons of the latent classes on environmental measures such as trauma exposure, socioeconomic indicators and social support (Table 1) showed no dramatic differences in these measures between the heavy and broad groups which might explain their divergence in alcohol-related problems (although all comparisons are statistically significant given the large sample size). Much work remains to understand the reasons – both genetic and environmental – for individual differences in the propensity to develop alcohol misuse.

## Conclusion

Overall, our recent study adds to the growing body of literature highlighting the complexity of the etiology and manifestation of alcohol misuse. Multidimensional deep phenotyping can be an innovative means to uncover some of the otherwise hidden genetic influences, although unravelling the biological mechanisms behind those influences is no trivial task. Our findings at both the phenotypic and genetic level point to the critical need to incorporate longitudinal data and developmental perspectives to push forward research of the genetic etiology of alcohol misuse.
